# Cytotoxicity Assessment and Indentation Size Effect of Low-Cost Experimental Implant Alloys

**DOI:** 10.1021/acsmaterialsau.6c00008

**Published:** 2026-04-09

**Authors:** Nompumelelo V. Nkosi, Divesha Essa, Amogelang C. Moalodi, Johnson Lawal, Yahya Choonara, Desmond Klenam, Michael O. Bodunrin

**Affiliations:** † Next Frontiers in Advanced Materials Laboratory, School of Chemical and Metallurgical Engineering, 37707 University of the Witwatersrand, Private Bag 3, WITS, 2050 Johannesburg, South Africa; ‡ Wits Advanced Drug Delivery Platform, Department of Pharmacy and Pharmacology, University of the Witwatersrand, Private Bag 3, WITS, 2050 Johannesburg, South Africa

**Keywords:** bioimplant, cytotoxicity, dislocations, indentation size effect, lightweight steels, strain gradient plasticity, titanium alloys

## Abstract

The development of affordable lightweight alloys with a combination of high mechanical strength and excellent biocompatibility is crucial for next-generation biomedical implants, particularly for middle- and low-income classes. This study investigates the relationship between processing, microscale mechanical behavior, and cytotoxicity of experimental titanium alloys (Ti-3Fe, Ti-4.5Al-1 V-3Fe, and Ti-6Al-1 V-3Fe) and a low-density stainless-steel (LDSS) alloy (Fe-20Mn-7Al-1C-3Cr-3Cu-3Mo), produced via casting and sintering. Their performance was benchmarked against those of commercial Ti-6Al-4 V and 316L stainless steel. Micro- and macro-indentation tests were conducted to evaluate the indentation size effect (ISE), and the Nix–Gao model was applied to quantify strain-gradient plasticity through the estimation of statistically stored dislocation (SSD) and geometrically necessary dislocation (GND) densities. In parallel, in vitro cytotoxicity was evaluated using NIH-3T3 fibroblast cells following the ISO 10993-5 guidelines. The experimental titanium alloys exhibited pronounced ISE behavior and the highest GND densities, indicating enhanced resistance to plastic deformation at the microscale compared to the LDSS alloys. Cytotoxicity results showed excellent biocompatibility for Ti-6Al-4 V and 316L and good compatibility for the experimental Ti–Fe alloys, with cell viability exceeding 70% at 100% extract concentration. In contrast, the LDSS alloys showed the lowest cell viability, with both the as-cast and sintered LDSS having a cell viability of less than 70%, thereby necessitating a detailed corrosion performance evaluation and further optimization. This performance was correlated with high Mn dissolution detected in the extract medium after the cytotoxicity assessment. The results demonstrate that experimental titanium alloys provide a promising pathway toward lightweight biomedical implant materials that combine microscale strengthening with acceptable biocompatibility.

## Graphical abstract

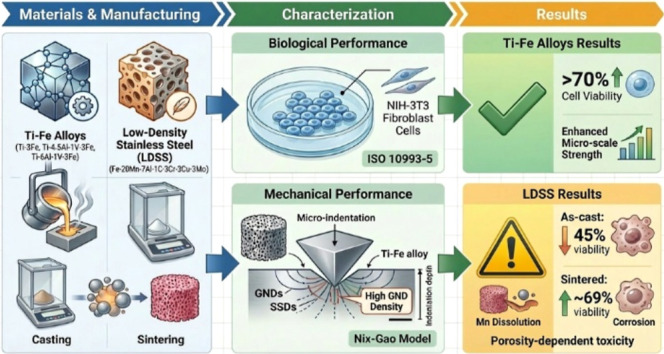

## Introduction

The increasing need for affordable biomedical implants has driven research into materials that can offer both mechanical reliability and biological compatibility at a reduced cost. Conventional implant metals, such as 316L stainless steel (SS) and titanium-based alloys, specifically Ti-6Al-4 V, have been widely used due to their high strength and excellent corrosion resistance, but their high cost or high density, mismatch in mechanical properties with human bone, and toxicity of alloying elements, resulting in infections, have prompted research for alternative materials.^1–4^

To develop affordable metallic implant materials, either the cost of titanium implants is reduced, or already affordable stainless steels are made lighter.^2,3^ Over the years, there have been efforts to reduce the cost of titanium alloys through modification of alloy chemistry, like replacing expensive alloying elements with cheaper ones that can fulfill similar functions,^2^ exploring alternative production routes like powder metallurgy or optimization of processing parameters. Using one or a combination of these approaches has led to a marginal reduction in the cost of titanium alloys without sacrificing the in-service performance of the alloys. For example, Ti-(4.5 or 6) Al-1 V-3Fe and Ti–Fe have been reported to have similar or slightly improved corrosion performance in simulated body fluids despite having up to 10% cost reduction in comparison with commercial-grade Ti-6Al-4 V.^5–8^ However, information regarding the cytotoxicity of these experimental alloys is unavailable. Hence, this study.

Lightweight steels developed by incorporating elements such as aluminum and manganese are emerging as promising candidates for the next-generation of affordable bioimplants, owing to their reduced density, low Young’s modulus, good corrosion resistance, and potential for improved biocompatibility if optimized.^1,9–12^

The concept of reducing the density of steel alloys for biomedical purposes is rooted in alloy design, where lighter elements like aluminum, magnesium, and silicon are introduced into the steel matrix.^9–12^ The development of lightweight steel was initially driven by the automotive industry’s need to reduce vehicle weight for improved fuel efficiency and lower emissions, with advanced high-strength steels and low-density alloys playing key roles in this effort.^1,4,9^ Building on this foundation, researchers have explored the use of these materials for biomedical applications where similar requirements for low density, high strength, and durability are critical.^4,13–16^

One of the main drawbacks for lightweight steels in biomedical applications is their corrosion resistance, which is generally lower than that of commercial stainless steels.^1,11^ The physiological environment that bioimplants are exposed to is highly corrosive, meaning that the implant material must resist both general and localized corrosion to prevent toxic ion release and adverse tissue reactions.^17,18^ Recent studies have shown that alloying strategies can enhance the performance of these steels, and specifically the addition of chromium, molybdenum, and copper can significantly improve the corrosion resistance of lightweight steels, leading to the development of lightweight stainless steels that combine low density with enhanced durability.^3,4,10,11,15,16,19–23^ Molybdenum is often added to increase pitting resistance by thickening the passive oxide layer,^13,21–23^ while copper is being explored for its antimicrobial properties. Copper additionally inhibits bacterial adhesion and biofilm formation through the release of Cu^2+^ ions, which inhibit bacterial cell membranes, generate reactive oxygen species, and interfere with specific bacterial enzymes thus killing the cells and hindering the formation of protective biofilms.^3,15,21^

The cytotoxicity assessment of lightweight bioimplant steels is essential prior to their clinical adoption, focusing on interactions with key cell types such as fibroblasts, which are crucial for tissue integration and healing. By comparing the cytotoxicity of lightweight steels with established materials like 316L SS and Ti-6Al-4 V, researchers aim to identify safer and more effective alternatives for biomedical implants.^2,24^ Thus, this study aims at assessing and comparing the cytotoxicity of 316L SS, Ti-6Al-4 V, experimental titanium alloys, and Fe–Mn–Al–C–Cr–Mo–Cu austenitic-based lightweight steels for bioimplant applications. The LDSS alloys and Ti–Fe alloys are developmental alloys for biomedical applications, and as such, cytotoxicity assessments have not been conducted on them in the past; thus, this study provides the foundation literature to show how close LDSS is to typical Ti-based biomedical alloys.

The indentation size effect (ISE) is an important phenomenon observed in small-scale hardness testing where the measured hardness of materials increases significantly as the indentation size decreases.^25^ This size dependence contradicts classical plasticity theories, which predict size-independent hardness because they lack intrinsic length scales.^26^ It has been demonstrated that the inclusion of strain gradient effects by accounting for gradients in plastic strain and the associated geometrically necessary dislocations can explain this behavior.^26^ The strain gradient plasticity model predicts that hardness increases notably when the indent size is comparable to or smaller than a characteristic material length scale, which varies with microstructural features.^25,26^

This effect is highly relevant for understanding the deformation behavior of materials at micrometer and submicrometer scales for biomedical implants where mechanical reliability and hardness at small scales are critical. The size-dependent response impacts how these materials perform under localized loading conditions and directly relates to their microstructural control and processing. By applying strain gradient plasticity theory, one can better interpret Vicker’s indentation test data to evaluate intrinsic material length scales, yielding insights into material strengthening mechanisms crucial for designing reliable biomedical implants with optimized mechanical properties.^25–29^

This study investigates the ISE in potential biomedical implant materials, comparing them to the currently used 316L SS and Ti-6Al4 V alloys to understand their deformation response at the microscale. Following the approach used in the study of strain gradient plasticity in carbonitrided steels,^25,27^ the Nix and Gao model is applied to analyze the relationship between hardness and indentation depth and the interaction of the indent with the dislocations in a material. The key objectives are to quantify and estimate the densities of Statistically Stored Dislocations (SSDs) and Geometrically Necessary Dislocations (GNDs), determine the characteristic material length scales, and identify the prevailing dislocation regimes (starvation, discrete, and continuum). This analysis aims to explain the fundamental strengthening mechanisms in the studied alloys, which are critical for predicting their mechanical performance and ensuring the reliability of biomedical implants under localized stress.

## Materials and Methodology

### Alloy Production

The commercial 316L SS and Ti-6Al-4 V alloys were procured as standard wrought products. The experimental low-density stainless steels (LDSS) with compositions based on the austenitic system (Fe-20Mn-7Al-1C-3Cr-3Cu-3Mo) were produced via two routes: powder metallurgy and traditional casting. For the powder metallurgy route, a spark plasma sintering machine was used to produce a 5 mm thick disc after mechanical alloying of constituent metallic powders for 12 h of mixing at 200 rpm and ball to High-purity (99%) elemental powders of Fe, Mn, Al, C, Cr, Cu, and Mo with angular morphology, procured from Sigma-Aldrich, were used as the starting raw materials. Sintering was carried out at a pressure of 50 MPa, a consolidation temperature of 1100 °C, and time of 5 min. The porosity of the sintered LDSS was reported by Mosoma et al, 2026, to be 2.38%, attributed to the sintering process.^30^ The experimental titanium alloys (Ti-3Fe, Ti-4.5Al-1 V-3Fe, and Ti-6Al-1 V-3Fe) were produced by a vacuum induction melting furnace followed by hot isostatic pressing treatment at a temperature of 920 °C and pressure of 120 MPa for 2 h to reduce porosity and breakdown as-cast structure.^5,8^ All samples were then sectioned, mounted, and metallographically prepared (ground and polished) to a mirror-like finish for subsequent microstructural, cytotoxicity assessment, and mechanical characterization.

### Microstructural Characterization

The titanium-based alloys were etched using a mixture of 100 mL of water, 3 mL of hydrofluoric acid, and 6 mL of nitric acid after metallographic preparation, while stainless steel-based alloys were etched using the V2A reagent to reveal the microstructures and characterized using an optical microscope. The optical micrographs of the etched alloy samples showing the (α + β) dual phase in titanium alloys and the dominant austenite phase in austenitic-based LDSS, respectively, are shown in Figure 1a–g.

**Figure 1:**
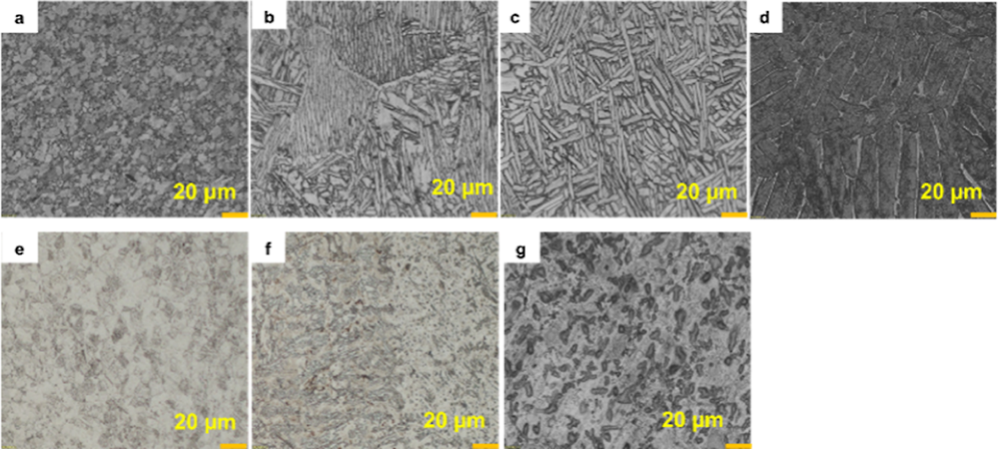
Classical microstructures of selected bioimplant alloys with (a) Ti-6Al-4 V, (b)­Ti-4.5Al-1 V-3Fe, (c)­Ti-6Al-1 V-3Fe, (d) Ti-3Fe, (e)­316L SS, (f) As-cast LDSS, and (g) Sintered LDSS.

### Cell Culture

The murine fibroblast cell line NIH-3T3 (Cellonex, Johannesburg, South Africa), was used to study in vitro cytotoxicity. Cells were cultured in the Roswell Park Memorial Institute (RPMI) medium supplemented with 10% fetal bovine serum (FBS), 100 U/mL penicillin, and 100 μg/mL streptomycin (Gibco, Thermo Fisher Scientific, Midrand, South Africa). Cultures were maintained at 37 °C in a humidified incubator with 5% CO_2_. The culture medium was replaced twice weekly. Upon reaching confluence, cells were detached from the culture flasks and subcultured at a 1:5 ratio.

### Sample Processing

Sample preparation and extraction procedures were initially performed following the protocol outlined by the National Library of Medicine and will be referred to as Method 1.^31^ The materials were machined into rectangular specimens in accordance with the ISO 10993 guidelines. Each sample was washed twice with double-distilled water, soaked for 24 h, and subsequently washed again using the same distilled water. The samples were then dried and sterilized by being autoclaved at 121 °C. The sterilized materials were used directly in subsequent experimental procedures.

All subsequent procedures were conducted under the aseptic conditions. Extraction media were prepared by incubating the test materials in serum-free RPMI medium at a ratio of 4 g per 20 mL, maintained at 37 °C in a humidified atmosphere containing 5% CO_2_ for 72 h. Each test and control group was prepared in triplicate, and the assay was performed in two independent runs. Upon reaching approximately 80% confluence, the culture medium was removed, and the cells were exposed to the extracted medium for 24 h. Quantitative analysis using method 1 was not possible due to the presence of contamination and particulate matter in the extracts.

A modified Method 2 was then carried out in the next batch of experiments. The samples were first rinsed with double-distilled water and then immersed in ethanol for 20 min. Following this, they were sterilized by autoclaving at 121 °C and subsequently washed twice with phosphate-buffered saline (PBS).^32^ Extraction was performed using complete RPMI medium (supplemented with 10% fetal bovine serum and 1% penicillin/streptomycin) at a ratio of 4 g of material per 20 mL of medium. The samples were incubated at 37 °C in a humidified atmosphere containing 5% CO_2_ for 24 h. After incubation, the extracts were collected and stored at 4 °C for further analysis^33^ using Atomic Absorption Spectroscopy (AAS).

### Cytotoxicity Assessment

Initially cells were seeded into 96-well plates at a density of 5000 cells per well and incubated for 24 h to allow attachment.^29^ Following incubation, the culture medium was replaced with 100 μL of alloy extracts prepared at four different dilutions (100, 50, 25, and 12.5). Culture medium without alloy extract served as the negative control, while medium containing 10% dimethyl sulfoxide (DMSO) was used as the positive control for cytotoxicity.

The cytotoxicity of the alloy samples was assessed using the MTT assay 3-(4,5-dimethylthiazol-2-yl)-2,5-diphenyltetrazolium bromide (Merck). The water-soluble MTT reagent penetrates viable cells and is enzymatically reduced by metabolically active cells to form water-insoluble formazan crystals. After 24 h of treatment the MTT reagent was added to each well containing samples and controls, and the reaction was allowed to proceed for 4 h. Subsequently, the insoluble formazan crystals were dissolved overnight. Absorbance was measured at 570 nm by using a microplate reader.

### Hardness Testing and Indentation Size Effect Theory

Microindentation testing was carried out using the FutureTech FM700 microhardness testing machine on mounted and polished surfaces. A Vickers indenter with a diamond tip was used at microhardness loads (0.3, 0.5, and 1) kgf and macrohardness loads (3, 5, and 10) kgf. A total of 15 indentations per load for each alloy were taken. The lengths of the indent diagonal (d_1_ and d_2_) were measured manually with the microscope attached to the indenter at a dwell time of 10 s.

The hardness (H) and (d_1_&d_2_) were recorded, and then the corresponding indentation depth (h) was calculated as shown in eq 1.

1 $$h=\frac{d}{2.\sqrt{2.}\tan\left(\frac{\theta }{2}\right)}$$

The data was analyzed using the strain gradient plasticity (SGP) mechanistic model developed by Nix and Gao, which relates the hardness (*H*) to the indentation depth (*h*), as shown in eq 2,^25–29^ ^,^ ^34–36^

2 $$H^{2}=H_{o}^{2}\left(1+\frac{h^{*}}{h}\right)$$

where *H*_o_ is the estimated macroscopic bulk hardness, representing the contribution from only the Statistically Stored Dislocations (SSDs) and h* is the characteristic length scale, a material constant indicative of the depth at which Geometrically Necessary Dislocations (GNDs) contributions become significant in the hardening of a material during indentation.

A linear plot of *H* ^2^ versus *1/h* was constructed from eq 2 and experimental data were obtained from the Vickers hardness test. The *y*-intercept of the linear fit gives H_o_^2^, and the slope gives the product of *H*_o_^2^ and h*, allowing both parameters to be determined. The macroscopic hardness value *H*_o_ was used to calculate the density of SSDs using the Taylor relation in eq 3.

3 $$\rho_{SSD}={\left(\frac{H_{o}}{3\sqrt{3}\alpha \mu b}\right)}^{2}$$

where α is the Taylor factor, μ is the shear modulus, and *b* is the magnitude of the Burgers vector (Ti-based alloys: *b* = 0.286 nm, μ = 44 GPa, and Austenitic Stainless-steel-based alloys: *b* = 0.255 nm, μ = 77 GPa).

The material length scale ( l̂ ) is used to determine the existence and magnitude of strain gradients to determine if they are large enough to contribute GNDs to flow stresses based on microindents scale regime^26^ and this is calculated as in eq 4.

4 $$\mathrm{l̂}=b{\left(\frac{\mu }{\sigma_{o}}\right)}^{2}$$

where σ_0_ is the material flow stress under conditions where there is no strain gradient.

The average density of GNDs, which accommodate strain gradients under the indent, was calculated using the relationship in eq 5. This equation was also used to estimate the average shear strain (γ) in the material by fitting the model to the experimental hardness-depth data and linked to the total dislocations’ density according to eq 6.

5 $$\rho_{GND}\approx \frac{4\Upsilon }{bh}$$

6 $$H\approx \mu b{\left[\rho_{GND}+\frac{4\Upsilon }{bh}\right]}^{1/2}$$

Finally, the total dislocation density responsible for the material’s strength was obtained by summing the contributions from both types of dislocations using eq 7. The spacing (L) between the dislocations was estimated according to eqs 8 and 9 for the SSDs and GNDs, respectively.

7 $$\rho_{T}=\rho_{SSD}+\rho_{GND}$$

8 $$L=\frac{1}{\sqrt{\rho_{SSD}}}$$

9 $$L=\frac{1}{\sqrt{\rho_{GND}}}$$

This methodology provides a comprehensive framework for deconvoluting the contributions of SSDs and GNDs to the observed strengthening, thereby linking the macroscopic indentation response to the underlying microscale dislocation mechanisms.

## Results

### Assessment of Sample Treatment and Extraction Methods

Visual and microscopic examination of cells following treatment using Method 1 revealed cloudy particulates, potentially resulting from corrosion contaminants from the alloy samples, as shown in Figures 2 and 3. These observations indicated that the initial processing and extraction procedures required modification to ensure extract clarity and experimental reliability, and thus the results could not be quantified for Method 1.

**Figure 2:**
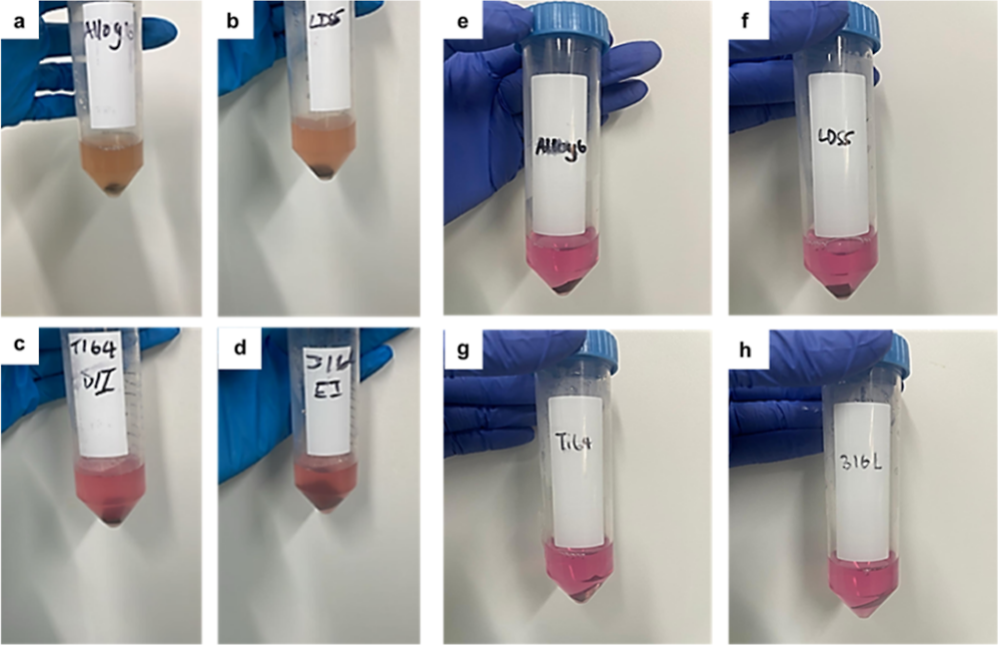
(a–d) Samples after 24 h extraction using method 1. (e–h) Alloy samples after 24 h extraction using Method 2.

**Figure 3:**
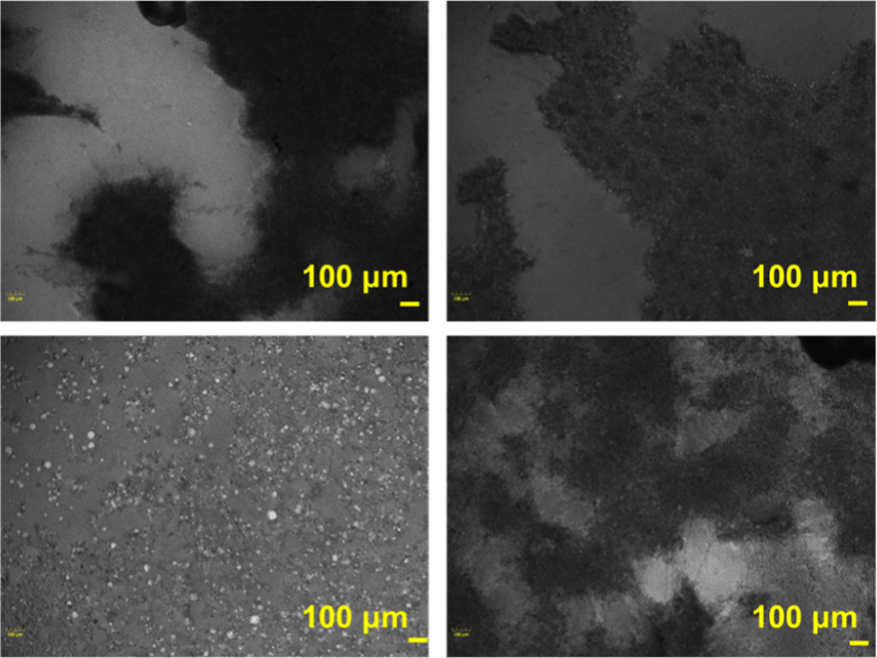
Representative images showing contamination of extracts prepared by Method 1.

Images of the samples were captured after a 24 h wash with double-distilled water. The LDSS sample exhibited visible signs of corrosion (Alloy 6: As-cast LDSS and LDSS: Sintered LDSS). Extracts prepared using sample treatment method 1 caused discoloration of the culture media, which was more pronounced in the LDSS samples in Figure 2a–d. In contrast, extracts from method 2 showed no observable color change in the media, as seen in Figure 2e–h.

The extract media were analyzed using atomic absorption spectroscopy to check for any corrosion products after the alloy samples were removed, and the compositions are summarized in Table 1. There was a substantial release of Mn from the LDSS media, as seen in Table 1. The as-cast and sintered LDSS showed Mn concentrations approximately 30 times higher than the control solution (approximately 1.1 mg/L vs 0.036 mg/L). This indicates active dissolution, likely from Mn-rich phases within these experimental steels, which is a critical factor for their biocompatibility assessment. Titanium was detected in all solutions, including the control solution. While the experimental titanium alloys (Ti-3Fe, Ti-4.5Al-1 V-3Fe, Ti-6Al-1 V-3Fe) showed relatively low Ti release (1.8–2.5 mg/L), the commercial Ti-6Al-4 V and, unexpectedly, the 316L SS showed higher values. The high Ti reading for 316L SS, which contains minimal titanium, suggests potential cross-contamination during sample preparation or analysis and needs to be investigated further. The 316L SS medium showed the highest levels of Cr and Fe, which is consistent with its composition and represents typical, moderate Cu ion release from a stainless steel. No other alloys showed dramatic Fe release. Copper levels were elevated across almost all samples, including controls. As Cu is not a declared alloying element in these materials, this points to systematic background contamination, possibly from laboratory reagents, tubing, or sample handling apparatus. The Al readings for the sintered LDSS, Ti-3Fe, and Ti-6Al-1 V-3Fe alloys were higher than the control, which aligns with their composition. This could be due to the dissolution of Al in the solution, which is negligible due to the low values. Proper and accurate corrosion experiments in the extracted media need to be carried out to ascertain the exact behavior of the solutions.

**Table 1:** Metal Concentration in Solution After Immersion, Measured by AAS

Solution (mg/L)	Control	As-cast LDSS	Sintered LDSS	Ti-6Al-4 V	316L SS	Ti-3Fe	Ti-4.5Al-1 V-3Fe	Ti-6Al-1 V-3Fe
Fe	0.27	0.32	0.28	0.28	0.35	0.23	0.21	0.13
Cr	0.63	0.61	0.59	0.75	0.83	0.44	0.51	0.57
Cu	1.22	1.49	1.30	1.54	1.68	1.11	0.98	1.05
Al	0.33	0.19	0.33	0.29	0.18	0.66	0.26	0.43
Mn	0.04	1.14	1.05	0.01	0.00	0.00	0.00	0.00
Ti	3.60	3.90	3.10	4.40	4.60	1.80	1.90	2.50

As shown in Figure 4, after soaking the experimental titanium alloys batch for 24 h in distilled water (4a) and 24 h extraction with RPMI culture media (4b) using method 2, there were no visible signs of corrosion contaminants from any of the alloys.

**Figure 4:**
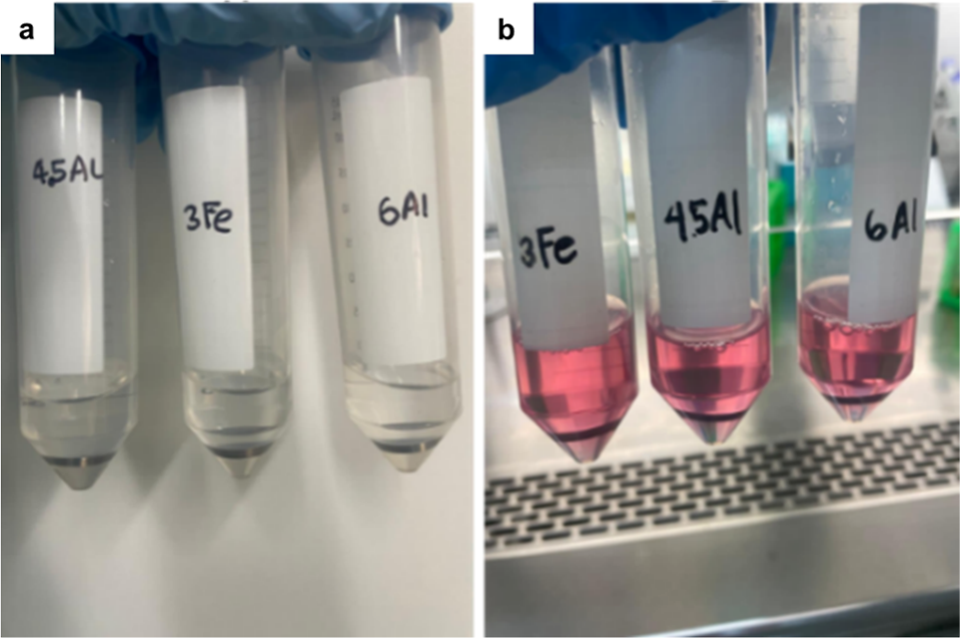
(a) Alloy samples after 24 h soaking in double distilled water; (b) After 24 h extraction with RPMI cell culture media.

### Microscopic Analysis of Seeded Cells

After seeding 5000 cells per well and incubating for 24 h, the cells reached approximately 90% confluence, leaving minimal space for further growth as seen in Figure 5a. Consequently, for the second sample treatment method, cells were seeded at a lower density of 2000 cells per well, as seen in Figure 5b, allowing clearer observation of cell morphology. Results were normalized within each experimental set, with the mean absorbance of the control group for each seeding density set as 100% viability. Treatment effects were calculated as a percentage of their respective density-specific control, and quantitative comparisons were restricted to ensure that the relative effects were independent of the initial cell number. The MTT assay was then performed following the same procedure described previously.

**Figure 5:**
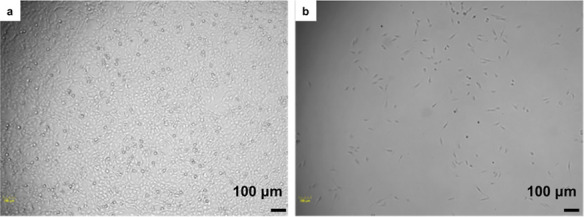
(a) Cells seeded at 5000 cells/well and (b) cells seeded at 2000 cells/well.

### Cytotoxicity Assessment

The absorbance values obtained from a microplate reader at 570 nm were directly proportional to cell viability and inversely related to the cytotoxicity of the samples. Figure 6 shows representative images of control and treated wells after 24 h of treatment with alloy sample extracts. The untreated control well (a.1) shows healthy attachment and elongated fibroblast morphology, while the toxicity control well (a.2) shows rounding of cells and detachment from the culture plate, indicating cell death. All the alloy treatments showed preliminary results for toxicity, with healthy cell morphology visible for all treatments, as well as varying degrees of cell rounding and lower density, indicating a degree of toxicity.

**Figure 6:**
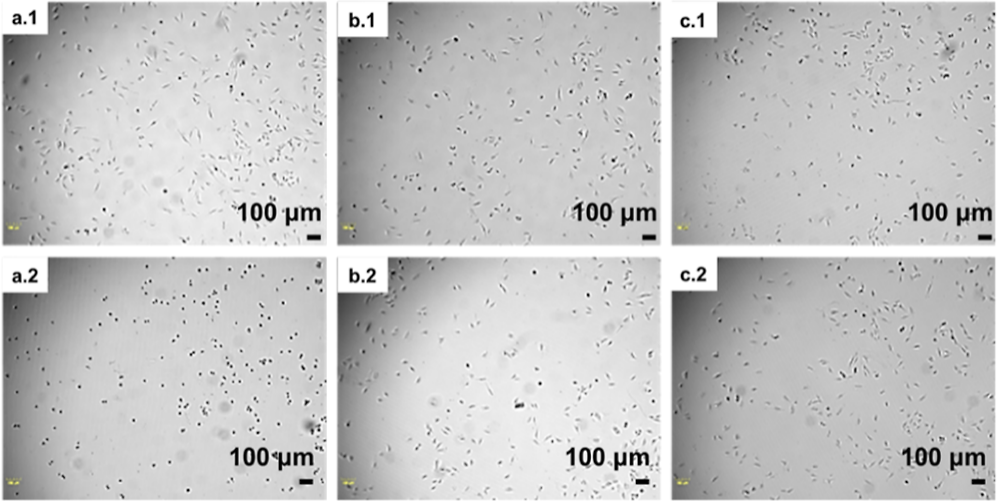
Sample images after 24 h of treatment. (a.1) Untreated negative control and (a.2) DMSO positive control for cytotoxicity. (B.1) As-cast LDSS, (c.1) Sintered LDSS, (b.2) Ti-6Al-4 V, and (c.2) 316L SS, all at 100% concentration.

The microscopy analysis showed that there were minimal to low toxicity effects of the alloy samples at 100% concentration (Figure 7) for the titanium experimental alloys. All extract treatments showed toxicity but at different degrees. Positive control shows rounded cells as dominant, while negative control shows elongated cells. The alloy extract treatment shows both but at varying degrees, as a function of dilutions and extract type. The characteristic elongated fibroblast morphology shown in the control group (a.1) was observed in all the sample-treated groups. Rounded morphology was observed in the DMSO treated group (a.2), demonstrating the toxic effect on the cells.

**Figure 7:**
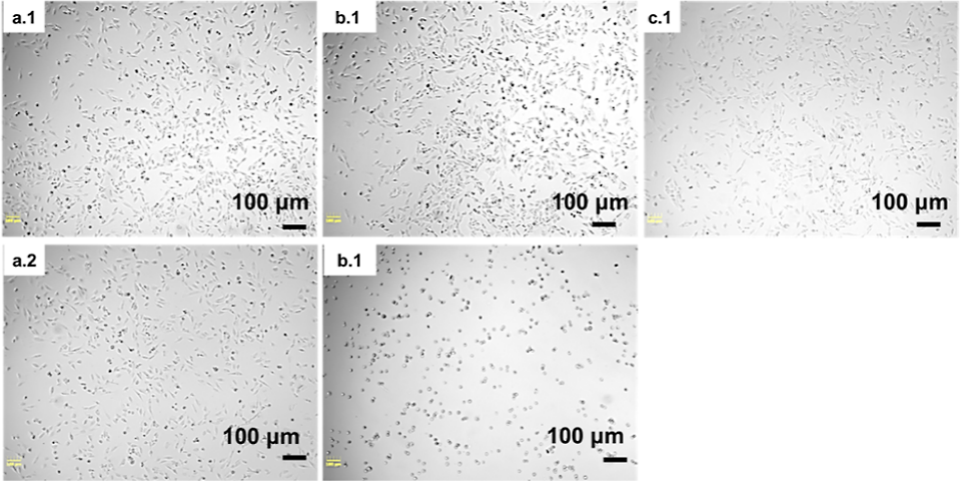
(a.1) Cells treated with culture media (negative control for cytotoxicity), (b.1) cells treated with Ti-3Fe, (c.1) cells treated with Ti-4.5Al-4 V-3Fe at 5000 cells/well, (a.2) cells treated with dimethyl sulfoxide (positive control for cytotoxicity), and (b.2) Cells treated with Ti-6Al-4 V–Fe.

The results for the MTT assay and cell viability of the treated cells with different concentrations are illustrated in Figures 8 and 9 for the different alloy samples analyzed per batch. Figure 8 illustrates the relationship between alloy extract concentration and the viability of NIH-3T3 cells for LDSS steels, as compared with commercial 316L SS and Ti-6Al-4 V. A clear dose–response is visible, with viability increasing as extract concentration decreases. The results showed dose-dependent toxicity across all sample experimental titanium alloys. At 100% concentration, the viability was >70%, while at concentrations of 50% or less, the toxic effects were negligible, with >95% viability. As-cast LDSS showed the lowest cell viability of 45%, followed by the as-sintered LDSS with a cell viability of 69%.

**Figure 8:**
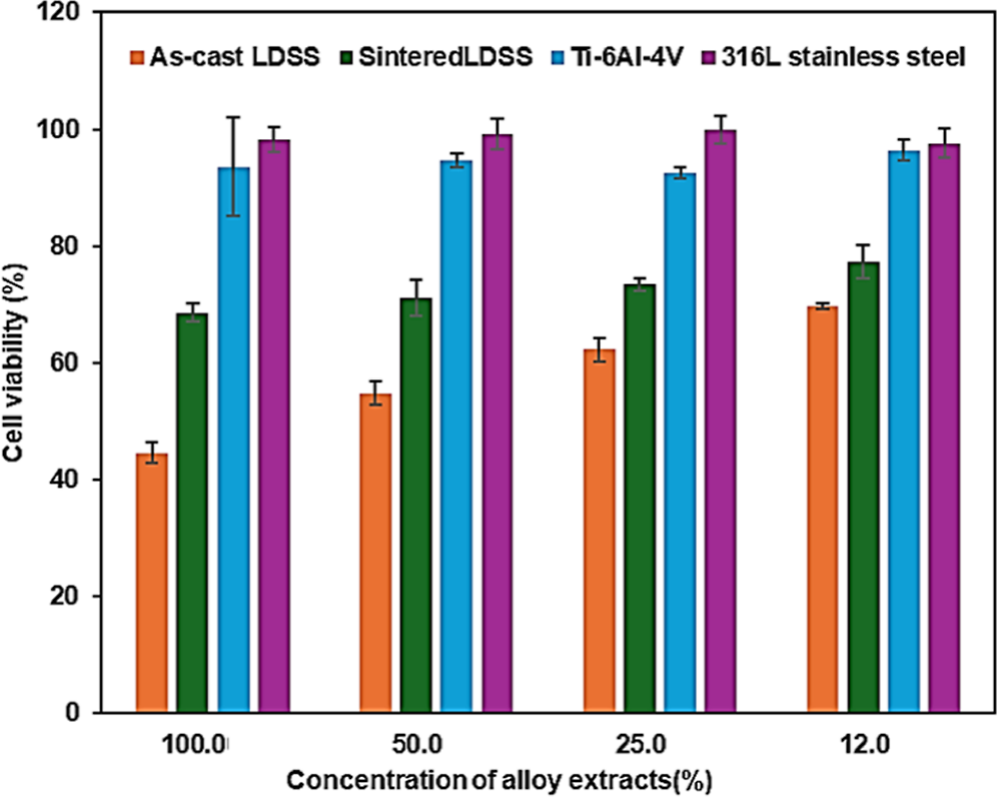
Graphical representation of cell viability for LDSS alloys as compared with commercial 316L SS and Ti-6Al-4 V.

**Figure 9:**
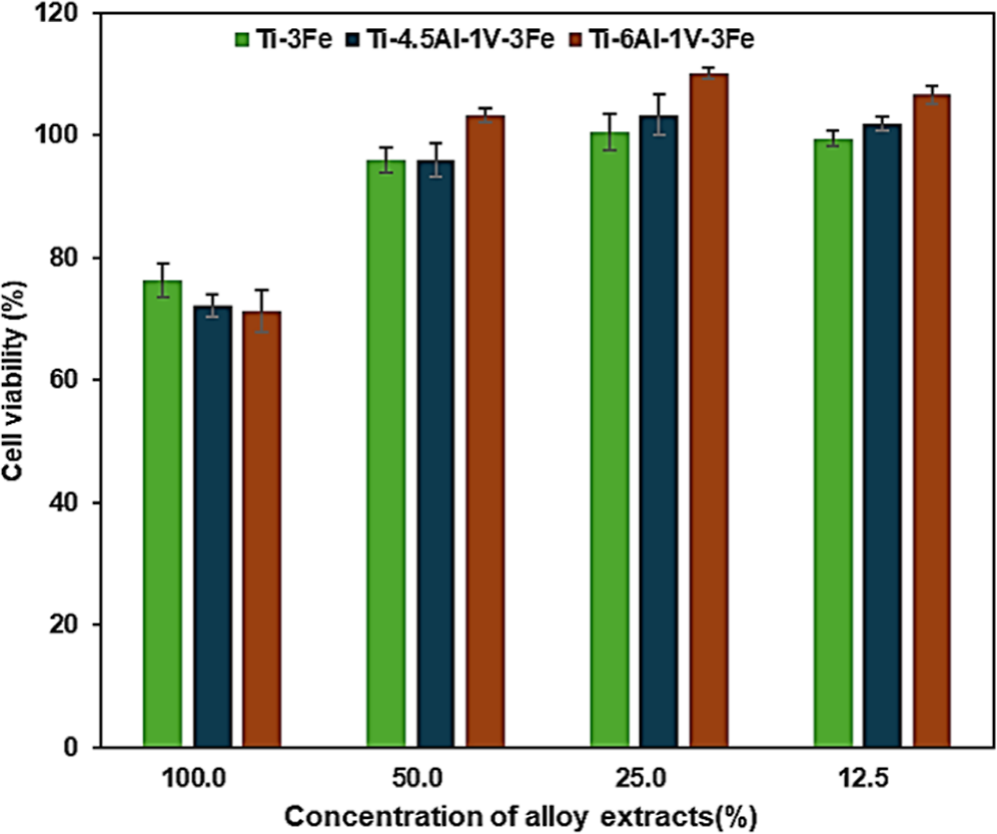
Graphical representation of the cell viability for experimental titanium alloys.

Figure 9 illustrates the relationship between the alloy extract concentration and the viability of NIH-3T3 cells. A clear dose–response is visible, with viability increasing as extract concentration decreases.

### Hardness Response to the Indentation Size Effect

The values obtained from the calculations using eqs 1–9 and based on the Nix–Gao Model are summarized in Tables 2 and 3. Table 2 presents the estimated average material macroscopic hardness (*H*_o_) (*y*-intercept of the plot on Figure 10b), material length scales (h*-the slope of Figure 10b) and SSD and GND spacings for the alloys using micro- and macro-indentation data. 316L SS had the lowest macroscopic hardness with a value of 0.94 GPa, while sintered LDSS exhibited the highest macroscopic hardness with a value of 3.34 GPa. 316L SS has the highest material length scale value of 18.09 μm, while Ti-3Fe has the lowest value of 4.32 μm. The high hardness of the LDSS is likely due to the composition of the alloys and the presence of brittle phases, as LDSS are brittle and hard in nature due to the formation of Al-intermetallic phases and k-carbide phases responsible for the strengthening.^10,11,15,30^ The measured Vickers hardness values of 316L SS using 6 indentations were in the range of typical literature values (approximately 140 HV) comparable to the bulk hardness estimated value of 0.94 GPa (≈96 HV), which is consistent with the average experimental measurements. The estimated low microhardness value of 316L SS might have also been attributed to the size of the sample, which was a thin plate, which was mounted and polished between indentation steps to allow for accurate hardness measurements.

**Table 2:** Estimated Average Material Dislocation Parameters for the Alloys

Alloy	*R* ^2^	*H*_o_ (GPa)	h* (μm)	ρ_SSD_ spacing (m)	ρ_GND_ spacing (m)
316L SS	0.81	0.94	18.09	3.52 × 10^–07^	8.66 × 10^–07^
Ti-6Al-4 V	0.84	2.93	13.45	6.85 × 10^–08^	7.76 × 10^–07^
Ti-3Fe	0.89	1.91	4.32	1.05 × 10^–07^	8.65 × 10^–08^
Ti-6Al-1 V-3Fe	0.78	3.15	10.82	6.36 × 10^–08^	7.65 E08
Ti-4.5Al-1 V-3Fe	0.68	2.91	9.53	6.90 × 10^–08^	7.83 × 10^–08^
As-cast LDSS	0.87	2.28	16.11	1.14 × 10^–07^	7.54 × 10^–08^
sintered LDSS	0.80	3.34	10.09	7.79 × 10^–08^	7.03 × 10^–08^

**Table 3:** Estimated Average Total Dislocation Densities for the Alloys

Alloy	ρ_SSD_(m^–2^)	ρ_GND_(m^–2^)	ρ_T_ (m^–2^)
316L	8.08 × 10^12^	1.33 × 10^14^	1.41 × 10^14^
Ti-6Al-4 V	2.13 × 10^14^	1.66 × 10^14^	3.79 × 10^14^
Ti-3Fe	9.02 × 10^13^	1.34 × 10^14^	2.24 × 10^14^
Ti-6Al-1 V-3Fe	2.48 × 10^14^	1.71 × 10^14^	4.18 × 10^14^
Ti-4.5Al-1 V-3Fe	2.12 × 10^14^	1.63 × 10^14^	3.75 × 10^14^
As cast LDSS	7.66 × 10^13^	1.76 × 10^14^	2.52 × 10^14^
Sintered LDSS	1.65 × 10^14^	2.02 × 10^14^	3.67 × 10^14^

**Figure 10:**
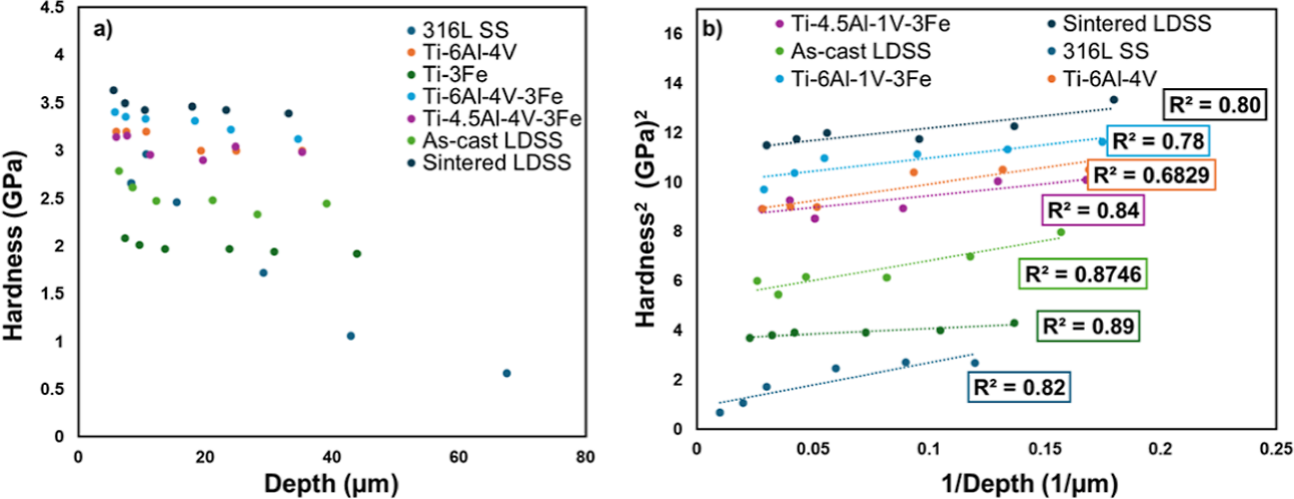
(a) Inverse relationship between hardness and depth. (b) Linear relationship between hardness squared and 1/depth for each alloy.

A summary of estimated average dislocation densities from eqs 1–9 for the alloys is given in Table 3.

Plots of hardness (*H*) versus the indentation depth (*h*) and hardness squared (*H* ^2^) versus the inverse of indentation depth (1/h), respectively, were drawn to determine the extent to which the Nix and Gao model relates the hardness and indentation size based on eqs 1–9.

Figure 10a shows the relationship between Vickers hardness and indentation depth, corresponding to applied loads from 0.3 to 10 kgf. All alloys exhibit the characteristic Indentation Size Effect (ISE), where hardness values decrease with an increasing indentation depth and load. This inverse relationship demonstrates enhanced resistance to deformation at smaller length scales in ranges of approximately 1–10 μm for these alloys,^36^ which is critical for understanding their micromechanical behavior in biomedical applications. Figure 10b demonstrates a linear dependence of H^2^ on 1/h, affirming that the ISE is consistent with the Nix-Gao model. This observation verifies that the increased material strengthening at the microscale is governed by the generation of GNDs due to strain gradients.^28,29^

The graphs plotted in Figure 11 show how the two types of dislocations behave differently for different alloys. The density of SSDs stays mostly the same at different depths. In contrast, the density of GNDs becomes much lower as the indentation depth increases. This difference shows that GNDs are mainly responsible for ISE, as they build up to handle the deformation of the material which is stronger at smaller scales.^25,34,35^

**Figure 11:**
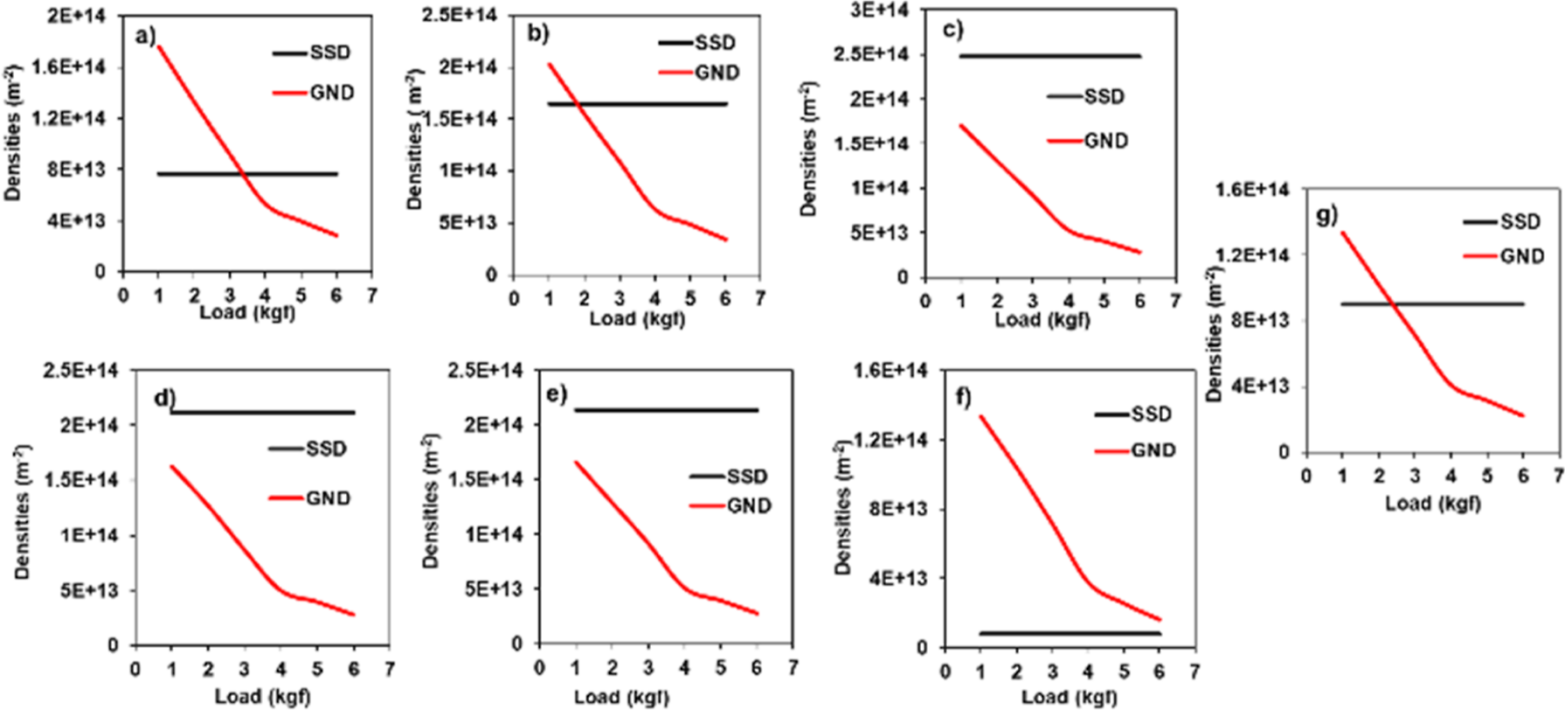
Comparison of statistically stored dislocation and geometrically necessary dislocation for different alloys: (a) As-cast LDSS, (b) sintered LDSS, (c) Ti-6Al-1 V-3Fe, (d) Ti-4.5Al-1 V-3Fe, (e) Ti-6Al-4 V, (f) 316L SS, and (g) Ti-3Fe.

The 316L SS alloy shows the strongest ISE effect followed by Ti-3Fe, while the sintered LDSS shows less sensitivity, followed by Ti-6Al-1 V-3Fe, as seen in Figure 10a,b.

The graph in Figure 12 shows how GND density changes with the indentation depth for all tested alloys. All alloys follow the same trend where the GND density is very high at small indentation depths and decreases rapidly as the indentation depth increases. This inverse relationship confirms that GND accumulation is depth-dependent and explains why these alloys appear harder when tested at smaller scales ISE.^36^ The strong linear relationship observed for all alloys confirms that ISE follows the Nix-Gao model. The steady increase in GND density with increasing 1/depth demonstrates that strain gradient effects become more dominant at smaller indentation scales.^34^

**Figure 12:**
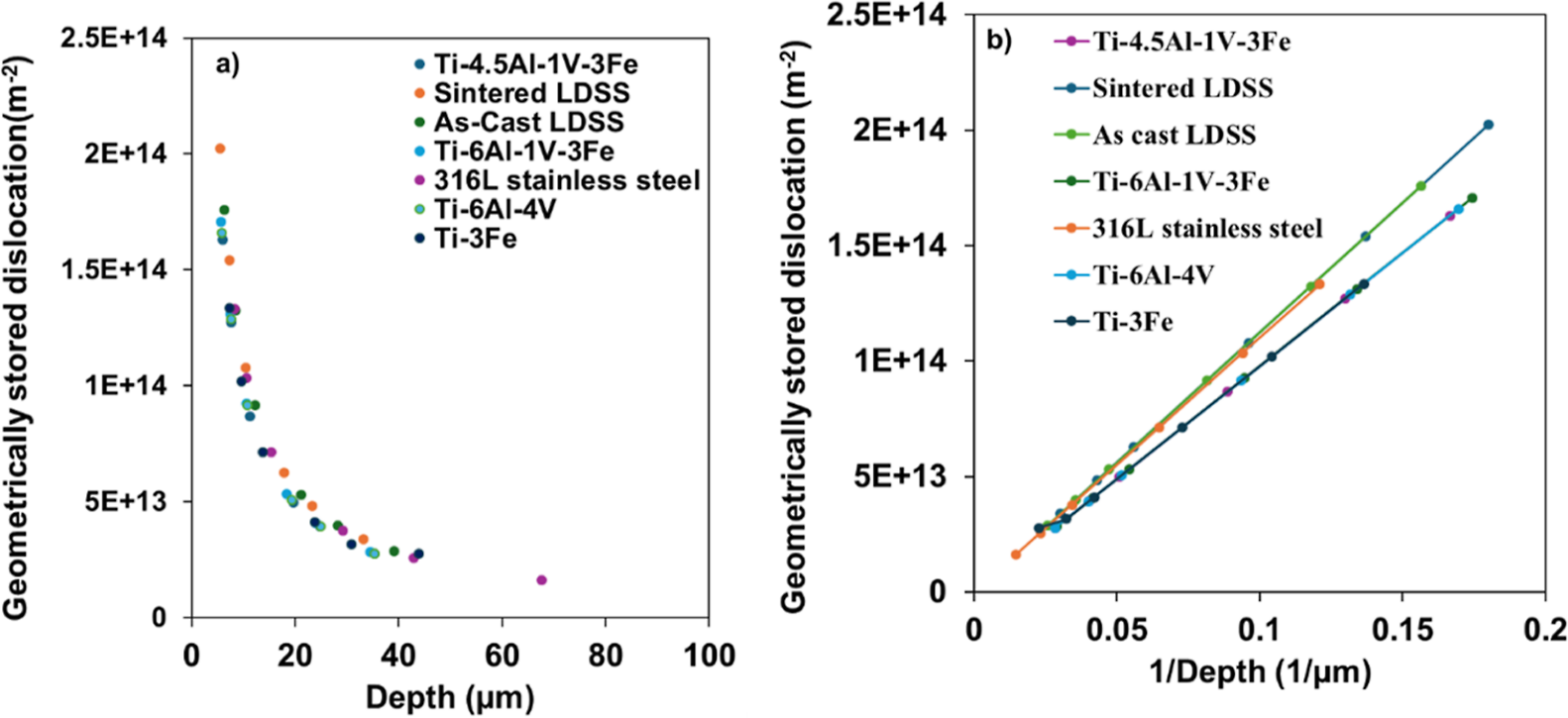
(a) Relationship between geometrically necessary dislocations and indentation depth. (b) Linear relationship between geometrically necessary dislocation and 1/depth.

## Discussion

### Cytotoxicity Assessment

The cytotoxicity assessment study provided a comprehensive understanding of how the tested alloys interact with mice fibroblast cells and how both alloy composition and production processing influence their biological performance when compared to commercial Ti-6Al-4 V and 316L SS. The MTT assay results demonstrated a clear dose-dependent relationship, where cell viability increased as the extract concentration decreased for all materials. This trend reflects the typical biological response of cells exposed to metallic extracts; the higher the dilution, the fewer ions or corrosion products in the medium/extract, and thus the less cellular stress is induced.^24,38,39^ In vivo, it is envisaged that the body’s continuous circulation of fluids would further dilute these ions, suggesting that the in vitro toxicity observed at high concentrations indicates a more extreme condition than is expected in the body, estimating their actual biological effect as evaluated in this study.^38,40^

The commercial Ti-6Al-4 V and the experimental titanium alloys (Ti-3Fe, Ti-4.5Al-1 V-3Fe, Ti-6Al-1 V-3Fe) all demonstrated high cell viability with values above 70%, which is the ISO 10993-5 threshold across all concentrations. This clearly indicates that these materials are noncytotoxic and are biocompatible. The Ti-6Al-4 V alloy, as expected, achieved cell viability near 94–96%, consistent with its long history of safe use in orthopedic and dental implants.^32,33^

The experimental titanium alloys also performed well maintaining 71–76% viability at 100% extract and exceeding 100% viability at 25% and 12.5% extracts, suggesting that not only were the cells viable but may even have thrived under low extract concentrations. The strong biocompatibility of these alloys can be attributed to the formation of a continuous TiO_2_ passive film on their surfaces which is chemically stable and resistant to corrosion.^5–8^ This oxide layer minimizes the release of metallic ions such as Fe^2+^ and V^5+^ which could otherwise trigger oxidative stress in cells.^2,41^

The small differences observed among the three experimental titanium alloys likely reflect variations in the microstructure and oxide film stability. Some studies have reported that the addition of Fe to titanium alloys may slightly increases the dissolution tendency of the β phase, while V stabilizes the passive oxide and enhances mechanical properties.^5–8,41–44^ This balance likely explains the moderate but still highly acceptable viability observed. These results correspond well with other Ti–Fe or Ti–V systems reported to exhibit high cyto-compatibility when Fe ≤ 5 wt % and high corrosion resistance.^5–8,24,31,32^ The main reason behind the slightly lower cell viability at 100% concentration needs further scrutiny given that the improved corrosion resistance of the experimental alloys was likely due to a thicker TiO_2_ film.

The 316L SS exhibited excellent cytocompatibility, with viability values above 97% at all concentrations. This aligns with its well-established medical use and performance due to its stable Cr-rich oxide layer, which prevents significant ion leaching.^22,43^

In contrast, both the LDSS alloys with the same composition and a Cr content of approximately 3 wt % exhibited a unique cell viability behavior likely due to the dissolution of Mn from the Fe–Mn–Al–C composition. Both alloy extract media analyses revealed a greater dissolution of Mn, which likely led to increased corrosion susceptibility of the alloys. Sintered LDSS displayed moderate cytocompatibility with viability around 69% at 100% extract, placing it near the borderline for acceptable biocompatibility. Meanwhile, the as-cast LDSS showed low cytotoxicity with only 44% cell viability at 100% extract and 55% at 50% extract concentration. This significant difference indicates that the casting and sintering processes detrimentally affect the surface quality and corrosion behavior with the release of ions in the simulated physiological environment. The as-cast alloy did not go through any homogenizing heat treatment, and this may have contributed to the low cell viability observed due to the casting defects and its heterogeneously distributed microstructure (Figure 1f) that may have promoted localized corrosion and Mn dissolution.

The microstructures of the alloys exhibit clear differences due to their distinct compositions and processing routes, as seen in Figure 1. The as-cast LDSS microstructure is characterized by a dendritic morphology typical of solidification from the liquid molten state. Elongated dendrites and interdendritic regions are evident in Figure 1f, indicating solute segregation during solidification and resulting in a relatively heterogeneous phase distribution. These dendritic features indicate directional growth during cooling and are commonly associated with as-cast alloys in the absence of postprocessing heat treatment or thermomechanical treatment. In contrast, the sintered LDSS microstructure displays a more equiaxed and homogeneous appearance, which is characteristic of materials produced through powder metallurgy. The microstructure consists of fine, irregularly shaped features that correspond to sintered powder particles formed through solid-state diffusion during sintering. Additionally, small residual pores can be observed, which are typical of sintered materials where full densification may not be achieved.

The porosity introduced during powder sintering likely exposed a large reactive surface area, allowing enhanced metal ion release, especially Fe, Mn, Cr, and Al, which are known to cause oxidative stress and damage to the mitochondrial activity in fibroblasts, thus contributing to a low cell viability.^16,32^ Sintering may promote internal oxidation and entrap alloying elements within oxide inclusions that later dissolve into the extract medium 2, increasing cytotoxicity.^24,41–44^ These oxides, together with the porous surface, act as active corrosion sites.

The higher concentration of Mn in the extract media shows a high Mn release in both as-cast and sintered LDSS with minimal differences due to the Fe–Mn–Al–C–Cr–Mo–Cu composition of both alloys (Table 1). Manganese is an essential trace element necessary for various physiological processes, but its effects on the human body can be both beneficial and harmful depending on the exposure levels.^45^ Maintaining a balance is crucial in the Mn content exposed to the human body. While it supports various physiological functions, excessive exposure can lead to severe health issues, particularly neurotoxicity. Understanding the mechanisms of Mn toxicity and monitoring exposure levels are vital for preventing adverse health effects. It has been reported in selected African countries that out of the 2.3–8.8 mg of Mn absorbed daily by the human body, only about 2.3 mg for men and 1.8 mg for women are needed daily,^46^ and thus careful control of Mn release in bioimplants should be studied when considering Mn-containing bioimplants.

The observed medium discoloration and particulate formation on the extract media for the as-cast and sintered LDSS alloys further supports active corrosion, as corrosion products and oxide debris may interfere with cell metabolism and reduce MTT absorbance readings and thus necessitates further investigation on the corrosion mechanism.^18^

When the improved preparation protocol (Method 2) was applied involving ethanol rinsing, phosphate-buffered saline washing, and autoclaving, no visible corrosion or extract discoloration was detected. This suggests that surface pretreatment is critical to remove residual contaminants and stabilize the oxide layer. Similar findings reported improved cell viability on titanium implants following sterilization and ethanol cleaning, attributing the improvement to the removal of surface residues and better oxide passivation.^32^

The results suggest that corrosion-induced ion release is the dominant factor controlling cytotoxicity rather than the bulk composition of the alloy. The higher surface-to-volume ratio and potential galvanic couples in the LDSS alloys could have accelerated electrochemical dissolution.^16,19^ A previous study demonstrated that microstructural stability and Cr content are critical in controlling corrosion and biocompatibility in Fe–Mn–Al-C steels.^16^

Summarily, the cytotoxicity results confirm that the Ti-based alloys are highly biocompatible and hold significant promise for affordable implant applications. Their combination of high mechanical strength and biological safety positions them as potential alternatives to Ti-6Al-4 V and 316L SS. Ultimately, these results reinforce the principle that biocompatibility is not an intrinsic property but a surface–driven interaction, governed by oxide chemistry, ion release, and microstructural integrity.^17^ The Fe–Mn–Al–C–Cr LDSS alloys require further optimization to fully understand the corrosion mechanism and the role of the alloying elements in influencing the biocompatibility of Fe–Mn–Al–C–Cr based alloys.

### Hardness Variation Due to the Indentation Size Effect

The alloys at microscale exhibited a significant Indentation Size Effect (ISE), where hardness values increased as the indentation depth and indent load decreased, as shown in Figure 10. This behavior is characteristic of small-scale plasticity and contradicts classical theories that do not include an intrinsic length scale.^27^ The observed ISE can be explained using strain gradient plasticity theory, which incorporates the influence of dislocations, namely, GNDs. These dislocations are essential to accommodate the steep strain gradients created by the indenter leading to additional hardening at shallow indentation depths.^26–29^

GNDS are dislocations generated during indentation or due to other strain gradients to prevent gaps, forming in the material, and these GNDs combine with statistically present dislocations existing in the material, the SSDs to form the hardness of a material.^36^ It has been reported that for larger indent sizes, the number of SSDs interacting with the indent is higher than the GNDs formed to accommodate the size and shape of the indent; however, for smaller indent sizes, the GNDs are more significant to leave space for the permanent deformation created by the indenter. The movement and generation of these GNDS leads to significantly higher hardness measured.^36^

The applicability of the Nix and Gao model is confirmed by the linear relationship between H^2^ and 1/h in the microindentation regime (Figure 10). From these plots, the bulk macroscopic hardness (*H*_0_) and the characteristic length (h*) were determined for each alloy. The experimental titanium alloys, especially Ti-6Al-1 V-3Fe and Ti-4.5Al-1 V-3Fe showed a steeper gradient in the relationship compared to the commercial Ti-6Al-4 V, indicating a more pronounced ISE and a greater contribution from GNDs to their strength.

Figure 11 shows a direct comparison of the SSD and GND densities. The experimental titanium alloys consistently demonstrated higher GND densities than their commercial counterpart due to the addition of Fe resulting in a higher BCC crystal structure. The BCC structure has more slip systems hence would show more plasticity than Ti-6Al-4 V with higher fraction of HCP.^2,24^ The strong correlation between GND density and the inverse of indentation depth as shown in Figure 12 is a classic sign of strain gradient plasticity.^25–27^ This relationship confirms that as the indentation volume shrinks, a higher density of GNDs is necessary to accommodate the plastic strain gradient leading to the observed increase in hardness.^25^ Figure 12b complements this by showing how the GND density changes with depth, balancing as the indentation depth enters a regime where strain gradients become less significant.

More pronounced indentation size effects in pure metals compared to alloys are coherent as alloys have additional mechanisms responsible for dislocation strengthening, such as precipitation strengthening and solid solution strengthening.^36^

It is imperative to study the effects of different processing techniques on length-scale parameters, like in the case of as-cast LDSS and sintered LDSS response to indentation and shear strain. The ISE behavior of these two alloys prepared differently is different and can be attributed to the effects of variations in shear strain on GNDs and dislocation generation.^25^ The differences in average shear strain may be due to various residual stresses leading to higher hardness values, as seen in the recorded data, more prevalent in sintered LDSS. This requires further investigation and is not within the scope of this work.

The primary reason for the observable differing mechanical behaviors can also be attributed to the different microstructural features, as shown in Figure 1. The titanium alloys have a dual-phase (α + β) structure, and the experimental alloys with their more refined phases provide a more effective barrier to dislocation movement.^2^ This facilitates a greater accumulation of GNDs as evidenced by their higher GND densities.^42^ In contrast, the austenitic single-phase structure of the LDSS offers good ductility but deforms through mechanisms like twinning, resulting in a lower capacity for GND storage^35–37^ and a less pronounced ISE compared to titanium alloys as shown in the hardness-depth profiles. The sintered LDSS with its inherent porosity disrupts the continuity of the metal matrix, thus affecting the mechanical properties detrimentally. These pores act as stress concentrators, preventing the uniform storage and distribution of GNDs,^22^ which explains its inferior performance relative to the as-cast version as shown in the dislocation density comparison in Figure 11.

The heterogeneity of alloys, in terms of phases and microstructural features, compared to pure metals plays a large role in determining the response of the interaction of the indent with dislocations; thus, a large sample size of hardness measurements is required to explain variations of individual measurements from the linear fit in the Nix and Gao model for all the alloys.

Localized loading of a bioimplant under service conditions refers to the situation where stresses and strains are not uniformly distributed across the implant during use in the body. Instead, highly concentrated stresses occur in specific regions due to geometry, loading mode, material mismatch, and biological environment. This concept is central to understanding the plasticity, failure, and long-term biocompatibility of implants. For long-term service, implants must satisfy two seemingly conflicting requirements: high strength, to resist fracture and wear, and sufficient plasticity (ductility), to accommodate local overload without catastrophic failure. Thus, controlled plastic deformation at the microscale is a design requirement for safe implants. The generation and accumulation of GNDs influences biocompatibility indirectly but critically by acting as corrosion initiation points and promoting metal ion release to the physiological environment. Thus, for LDSS alloys, the excessive segregation of Mn or GND localization can compromise the longevity of the bioimplant.

At present, there is insufficient evidence to fully explain the observed variations in cell viability for the LDSS alloys in comparison to those of the Ti experimental alloys. However, the differences in the AAS extract composition results and manganese concentrations in the extract solutions obtained after cytotoxicity testing indicate that further optimization is required for processing these alloys, which may provide useful insight into the underlying mechanisms.

## Conclusions

This study demonstrates that the newly developed experimental alloys, particularly experimental titanium alloys, hold significant promise for biomedical implant applications.

- The experimental titanium alloys show excellent biocompatibility when exposed to physiological environments with concentrations of less than or equal to 50 percent fibroblast cells, and they show enhanced resistance to plastic deformation at the microscale, evidenced by the indentation size effect.
- The experimental as-cast and sintered low-density stainless steels with a cell viability of less than 70% revealed mild cytotoxicity and require further investigation.
- In comparison, the best-performing experimental titanium alloys reliably outperformed commercial materials such as 316L SS and Ti-6Al-4 V in terms of microscale strengthening and dislocation mechanisms. This suggests that these experimental alloys could pave the way for developing next-generation implants that are lighter, stronger, and just as safe as current options.

Future work will investigate different models to fit the hardness data and evaluate the strain gradient plasticity. Furthermore, research will continue the development of these alloys to improve the surface properties of these alloys through methods such as homogenizing heat treatments, anodization, and the application of bioactive coatings, with the goal of increasing their corrosion resistance. Once optimized, these materials will need to undergo thorough testing in animal models to assess their long-term biosafety, biocompatibility, and capacity for osseointegration prior to any consideration for clinical use. Further corrosion and mechanical assessments on the alloys will also be done to further understand the effect of composition, microstructure, and processing parameters on the LDSS alloys.
